# Altered Composition of the Oral Microbiota in Depression Among Cigarette Smokers: A Pilot Study

**DOI:** 10.3389/fpsyt.2022.902433

**Published:** 2022-07-19

**Authors:** Mohammad Tahseen Al Bataineh, Axel Künstner, Nihar Ranjan Dash, Rushud Mahmood Abdulsalam, Rafla Zaid Ali Al-Kayyali, M. Besher Adi, Habiba S. Alsafar, Hauke Busch, Saleh Mohamed Ibrahim

**Affiliations:** ^1^College of Medicine, University of Sharjah, Sharjah, United Arab Emirates; ^2^Department of Genetics and Molecular Biology, College of Medicine and Health Sciences, Khalifa University of Science and Technology, Abu Dhabi, United Arab Emirates; ^3^Center for Biotechnology, Khalifa University of Science and Technology, Abu Dhabi, United Arab Emirates; ^4^Lübeck Institute of Experimental Dermatology, University of Lübeck, Lübeck, Germany; ^5^Institute for Cardiogenetics, University of Lübeck, Lübeck, Germany; ^6^Department of Biomedical Engineering, Khalifa University of Science and Technology, Abu Dhabi, United Arab Emirates; ^7^Department of Mathematics, Khalifa University of Science and Technology, Abu Dhabi, United Arab Emirates

**Keywords:** avoidance, activation, BADS, metagenomics, oral microbiome

## Abstract

Alterations in the oral microbiota composition may influence mental health. However, linkages between compositional changes in the oral microbiota and their role in mental health among cigarette smokers remain largely unknown. In this study, we used shotgun metagenomics data for the oral microbiome of 105 participants. The data showed *Bacteroidota, Fusobacteriota, Firmicutes, Proteobacteria*, and *Actinobacteria* to be the most abundant phyla; *Streptococcus, Haemophilus D*, and *Veillonella* are the most abundant genera. Then, we clustered our subjects into avoidance and activation groups based on the behavioral activation for depression scale (BADS). Interestingly, the avoidance group exhibited a higher oral microbiome richness and diversity (alpha diversity). Differential abundance testing between BADS avoidance and activation groups showed the phyla *Bacteroidota* (effect size 0.5047, q = 0.0037), *Campylobacterota* (effect size 0.4012, q = 0.0276), *Firmicutes A* (effect size 0.3646, q = 0.0128), *Firmicutes I* (effect size 0.3581, q = 0.0268), and *Fusobacteriota* (effect size 0.6055, q = 0.0018) to be significantly increased in the avoidance group, but *Verrucomicrobiota* (effect size−0.6544, q = 0.0401), was found to be significantly decreased in the avoidance risk group. Network analysis of the 50 genera displaying the highest variation between both groups identified *Campylobacter B, Centipeda*, and *Veillonella* as hub nodes in the avoidance group. In contrast, *Haemophilus* and *Streptococcus* were identified as hub nodes in the activation group. Next, we investigated functional profiles of the oral microbiota based on BADS avoidance and activation groups and found Lysine degradations pathway was significantly enriched between both groups (ANCOM-BC, q = 0.0692). Altogether, we provide evidence for the presence of depression-related changes in the oral microbiota of smokers and possible functional contribution. The identified differences provide new information to enrich our understanding of oral microbiota-brain axis interplay and their potential impact on mental health.

## Introduction

The oral microbiome is a collective genome of microorganisms that reside in the oral cavity and their interactions with the host. Recent clinical studies have suggested an intriguing linkage between the microbiome of the gut and the central nervous system known as the gut-brain axis that influences the host's mental functions and behavior (1). The oral microbiome primarily consists of phyla *Actinobacteria, Bacteroidetes, Chlamydiae, Firmicutes, Fusobacteria, Proteobacteria*, and *Spirochaetes* that influence oral health. Microbiome alterations due to changes in diet, aging, immune status, and many other factors have been linked to common mental health disorders such as depression and anxiety (2–5). These factors play a significant role, in addition to smoking, in the microbiota complexity. Underscoring the need to control for these confounders during the analysis.

The oral microbiome constantly changes in response to the host's lifestyle, including cigarette smoking (6, 7). Smoking is known to cause deterioration in oral microbial ecology, known as dysbiosis, leading to an array of local and systemic pathologies (8–10). Smoking causes significant depletion of the phylum *Proteobacteria* and the genera *Capnocytophaga, Peptostreptococcus*, and *Leptotrichia* and enrichment of the phyla *Firmicutes* and *Actinobacteria*, as well as of the genera *Atopobium, Streptococcus* in the oral cavity when compared to people who never smoked. Furthermore, among smokers, the oral microbiome composition differs between current and non-current smokers; a noticeable difference is also seen whether the non-current smokers were former smokers or those who have never smoked (11, 12). Interestingly, the oral microbiome tends to restore its abundance following smoking cessation, although the duration and trend may vary significantly (13).

Smoking affects people's mental health, although the relationship between the two is still unclear. Smoking rates amongst those diagnosed with mental illnesses are higher than in the general population. It is linked mainly with anxiety, depression, schizophrenia, and panic disorder (14). Adults with depression are twice as likely to smoke as adults without depression. Most people start to smoke before showing signs of depression, so it is unclear whether smoking leads to depression or depression encourages people to start smoking (15). Currently, several hypotheses have been proposed to explain the high rates of smoking in people with depression and anxiety, including self-medication (16). However, the literature still lacks information regarding smoking-related oral microbiome alteration and its association with mental health and disease conditions such as depression.

In order to decipher our understanding of the link between smoking, oral microbiome, and depression, we conducted metagenomic profiling of the oral microbiome composition of 105 healthy individuals from Middle Eastern communities.

## Materials and Methods

### Study Design

We evaluated individuals from local communities in the United Arab Emirates who smoked cigarettes for over 5 years and non-smokers that were otherwise healthy, excluding those with chronic respiratory illness, reported antibiotic use, and prescribed probiotic use for the past 3 months.We have also assessed the behavioral activation for depression scale (BADS), as previously described (17). Briefly, this scale contains 9 items with scores ranging from 0 to 54, with high total scores representing higher activation while a high score on the avoidance subscale means more avoidance. We also collected buccal swabs from all participants using Isohelix DNA/RNA Buccal Swabs (Isohelix Ltd. Harrietsham, United Kingdom) following the manufacturer's instruction (Isohelix Ltd.). Participants did not eat, drink, smoke, clean their teeth, or chew gum for the past 1–3 h prior to collecting the samples. Furthermore, participants were asked to rinse their mouths with water just before sample collection. Samples where then transferrdinto a sterile container, stored immediately in liquid nitrogen, and then transferred to a – 80°C freezer. All participants in the study signed written informed consent, and the Research Ethics Committee at the University of Sharjah approved the study (REC-18-02-18-01).

### DNA Extraction and Sequence Processing

DNA was extracted using the Qiagen MagAttract PowerSoil DNA KF Kit (Formerly MO Bio PowerSoil DNA Kit) using a KingFisher robot. DNA quality was evaluated visually via gel electrophoresis and quantified using a Qubit 3.0 fluorometer (Thermo-Fischer, Waltham, MA, USA). Libraries were prepared with the Illumina Nextera library preparation kit using an in-house protocol (Illumina, San Diego, CA, USA). Paired-end sequencing using NextSeq 500 followed by shotgun metagenomic with the Sunbeam pipeline as described before (10). Raw sequencing reads were submitted to ENA and are available under accession number PRJEB53577.

### Statistical Analysis

All analyses were performed in R (v4.1.0). If necessary, *p*-values were corrected for multiple testing using Benjamini-Hochberg correction and are presented as q-values (18). For non-parametric testing of two groups, Wilcoxon tests were used and for contingency tables, X^2^-tests were applied with 1,000 replicates to compute *p*-values using Monte Carlo simulation. For analysis and visualization, the following additional R packages were applied: tidyverse (v1.3.1) for data handling; ggplot2 (v3.3.6), ggpubr (v0.4.0), and patchwork (v1.1.1) for visual presentation of the results. Additional R packages and further information about data analysis are given below in the subsections.

### Data Processing and Quality Assessment

Already processed sequencing data from Pwas imported as phyloseq object [phyloseq v1.36.0 (19)] in R v4.1.0 and operational taxonomic units occurring in less the 20 individuals with just 1 contig were removed (2,292 OTUs left after filtering) (10). For further analysis, data was summarized at genus level using the *phyloseq::tax_glom* function.

### Alpha and Beta Diversity

Alpha diversity on genus level was estimated on non-normalized counts using DivNet v0.3.7 (sample-wise Shannon diversity; OTU6740 selected as base taxon) to account for observed and unobserved taxa. Differences in alpha diversity between groups were calculated using the *betta_random* function (breakaway v4.7.2) (20, 21) with BADS group (activation or avoidance) as corresponding fixed effects and gender as random effect to adjust for gender differences. To assess beta diversity on genus level, data was centered log-ratio (*clr*) transformed and distances were calculated using Euclidean distance (Aitchison distance). Permutational multivariate analysis of variance using distance matrices (PERMANOVA) was used to analyze differences in beta diversity (*adonis* function, as implemented in the vegan package v2.5-7, with 99,999 permutations and BADS group as corresponding fixed effect).

### Differential Abundance Testing

Differentially abundant taxa between BADS groups (model: *H* ~ *BADS_group*) were identified using Analysis of Compositions of Microbiomes with Bias Correction (ANCOM-BC v1.6.0; zero_cut = 0.8, tol = 10x10^−6^, max_iter = 10,000, struct_zero = TRUE, Benjamini-Hochberg correction to correct for multiple testing) (22). Beta coefficients (effect sizes) and standard errors were obtained from the ANCOM-BC log-linear model and were used for visualization. To verify ANCOM-BC results and account for potential gender effects, a count regression for correlated observations with the Beta-binomial approach implemented in corncob (v0.2.0) was applied with gender in the NULL model (*H*_0_ ~ *gender*) to account for gender effects (23). The full model comprised gender and BADS group to test for differential abundant taxa (*H*_1_ ~ *BADS_group* + *gender*). Resulting *p*-values were corrected for multiple testing using Benjamini-Hochberg correction.

### Network Analysis

Network reconstruction on *clr*-normalized genus abundances was performed using the SparCC approach (24), as implemented in the R package NetCoMi (v1.0.2). For this analysis, the 50 genera with the highest variance between groups were selected. Significant edges were selected using Student's *t*-test and community structures were estimated using greedy optimization of modularity. Hub node detection was performed using a threshold of 0.8, and a quantitative assessment of the network was performed using a permutation approach (100,000 bootstraps) with an adaptive Benjamini–Hochberg correction to adjust *p*-values for multiple testing.

### Evaluation of Functional Profiles

Previously inferred functional profiles were imported into R as phyloseq object (10). Alpha diversity was estimated on subsampled data (*phyloseq::rarefy_even_depth* function with seed set to 1 and without replacement; 3,000 counts per sample) using Shannon index (*diversity* function; vegan package v2.6-2). Beta diversity on the full data was estimated as described above for the sequencing data, and differential abundant functional profiles were identified using ANCOM-BC (described above).

## Results

### Bacterial Analysis and Taxonomic Composition Showed Significantly Higher Alpha Diversity Among the BADS Avoidance Risk Group

This study assessed buccal swabs from 105 healthy individuals sampled from the local communities in correlation with their cigarette smoking status, among other criteria such as BADS. We further divided participants based on BADS into avoidance and activation groups. We calculated 53 with more avoidance scores and 52 with higher activation, all matched for age, gender, and BMI (Table 1).

**Table 1:** Demographics of study cohort.

**Characteristic**		**Avoidance BADS (*n* = 53)**	**Activation BADS (*n* = 52)**	***p*** **-value**	**Test**
Age (in years)	mean, range	31.04 (21–62)	29.65 (21–49)	0.6682	Wilcoxon
Gender	M, F%	92.5, 7.5	90.4, 9.6	0.7403	X^2^-test^**^
Ethnicity				0.4605	X^2^-test^**^
MENA		52.83	57.69		
Asians		16.98	23.08		
Africans		20.75	15.38		
American		3.77	1.92		
European		5.66	1.92		
BMI	Mean, IQR	25.18 (19.08–31.28)	24.71 (18.23–31.19)	0.6284	Wilcoxon
Exercise	in %	79.25	51.92	0.4266	X^2^-test^**^
Animal exposure	in %	20.75	13.46	0.4146	X^2^-test^**^
Smoking	in %	49.06	55.77	0.5774	X^2^-test^**^
BADS	Mean, s.d	37.45 (+/−6.11)	24.42 (+/−4.29)	*p* < 0.001	Wilcoxon

*MENA, Middle East and North African; BMI, Body mass index; IQR, Inter-quartile range; BADS, Behavioral Activation for Depression Scale*.

** *X^2^-tests: 1,000 bootstraps to compute p-values by Monte Carlo simulation*.

For sequencing analysis of the oral microbiome, we aggregated taxa abundances at phylum and genus level and plotted the relative abundances of all phyla and the most abundant genera (abundance >5%, Supplementary Figure 1).

**Figure 1:**
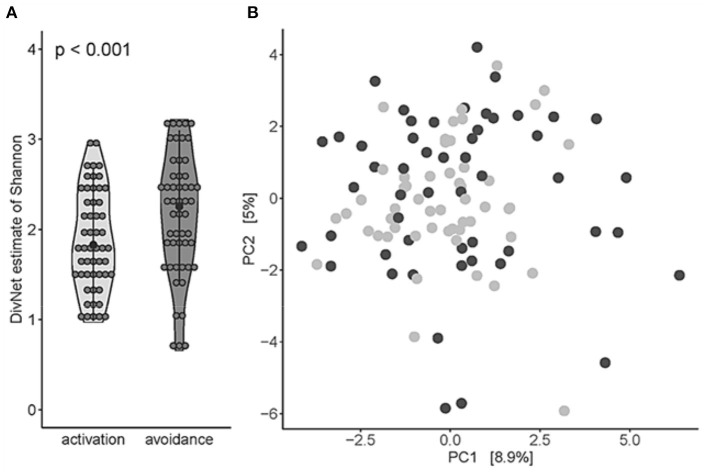
Oral microbiota of the BADS avoidance group shows a higher level of diversity based on BADS*. **(A)** Alpha diversity (Shannon) on genus level was significantly different between both groups (*betta test, p* < 0.001). **(B)** Beta diversity estimated using Aitchison distance for the community composition at genus level (PERMANOVA, *p* = 0.06638, *R*^2^ = 0.0120, 99,999 permutations); light gray refers to avoidance group, dark gray to activation group, respectively.

*Bacteroidota, Fusobacteriota*, and *Firmicutes, Proteobacteria*, and *Actinobacteria* were found to be the most abundant phyla, and the most abundant genera were *Streptococcus, Haemophilus D*, and *Veillonella*. Next, we estimated the alpha diversity of the oral microbiome between BADS avoidance and activation groups using Shannon's diversity index. The BADS avoidance risk group showed significant higher diversity (*betta test* with gender as a random variable to correct for gender effects; *p* < 0.001, estimate = 0.2935 ± 0.0850; Figure 1A). Community composition at genus level (beta diversity estimated using Aitchison distance) was not significantly different between BADS groups (PERMANOVA; *p* = 0.0664, *R*^2^ = 0.0120; 99,999 permutations; Figure 1B).

### Differential Bacterial Abundance Testing Between BADS Avoidance and Activation Groups Shows That *Bacteroidota, Campylobacterota, Firmicutes A, Firmicutes I*, and *Fusobacteriota* to Be Significantly Increased in the Avoidance Risk Group

**Figure 2:**
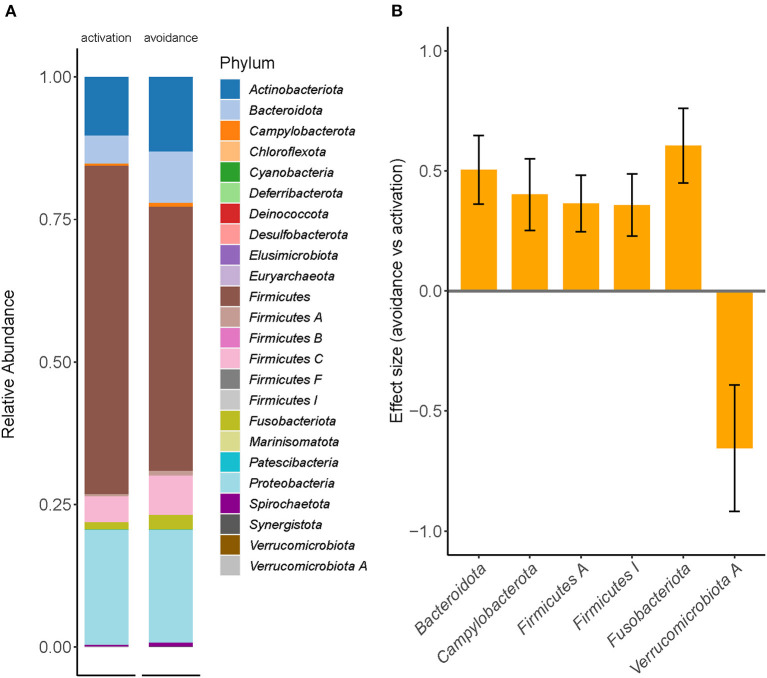
Differential abundance testing revealed significant enrichment differences between BADS avoidance and activation groups. **(A)** Relative taxonomic abundances are shown for phylum and summarized per group (mean abundance). **(B)** Two phyla were found to be significantly increased in the avoidance group (ANCOM-BC, q <0.05); whiskers denote standard deviation.

To further explore compositional differences between both groups, we conducted a differential abundance analysis using a compositional data approach with bias-correction (ANCOM-BC) to identify taxa with significantly different abundances between the avoidance and the activation group (Figure 2A).

The phyla *Bacteroidota* (effect size 0.5047, q = 0.0037), *Campylobacterota* (effect size 0.4012, q = 0.0276), *Firmicutes A* (effect size 0.3646, q = 0.0128), *Firmicutes I* (effect size 0.3581, q = 0.0268), and *Fusobacteriota* (effect size 0.6055, q = 0.0018) were found to be significantly increased in the avoidance risk group, but *Verrucomicrobiota* (effect size −0.6544, q = 0.0401), was found to be significantly decreased in the avoidance risk group (Figure 2B), whereas no genera were found to be significantly different between the groups. We applied a second approach using count-regression for correlated observations (corncob) to validate our findings further to determine the differentially abundant taxa between BADS avoidance and activation groups after correction for gender effects (Supplementary Figure 2).

**Figure 3:**
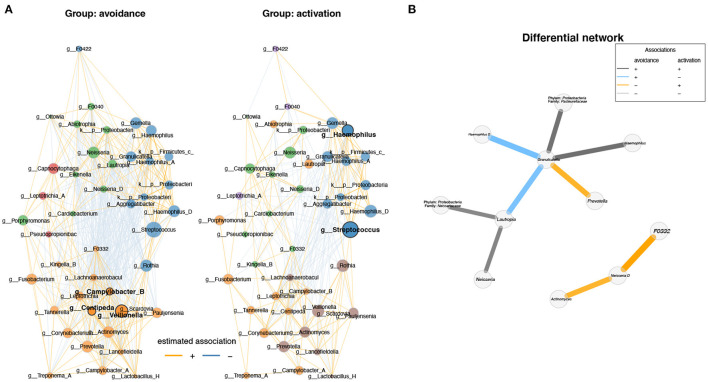
Differential network analysis between BADS avoidance and activation groups shows different correlations between genera. **(A)** Network analysis of the top 50 genera showing the highest variation between the two conditions and visualized the results (degree, betweenness centrality, closeness centrality; p > 0.05). Network specific hub nodes are shown in bold font. **(B)** The differential network shows the different correlations between genera (Fisher exact test, p-adj < 0.1 BH adjusted); if genus annotation was not available, phylum and family are shown.

Besides the phyla identified in Figure 2, eight additional phyla were found to be significantly between the two groups (q < 0.01; Supplementary Figure 2A). On genus level, 40 genera showed significant differences between the two groups (q < 0.01; Supplementary Figure 2B). Next, we sought to explore the difference between both conditions using differential network analysis. Network analysis of the top 50 genera displayed the highest variation between the two conditions (Figure 3A).

Network properties are not significantly different between both conditions in terms of degree, betweenness centrality, and closeness centrality (*p* > 0.05). However, we found that each network has specific hub nodes. *Campylobacter B, Centipeda*, and *Veillonella* were identified as hub nodes in the avoidance group, whereas *Haemophilus* and *Streptococcus* were identified as hub nodes in the activation group (Figure 3B).

### Functional Analysis of Oral Microbiota Reveals Significantly Different Abundance of Lysine Degradations Among Others Based on BADS Avoidance and Activation Groups

**Figure 4:**
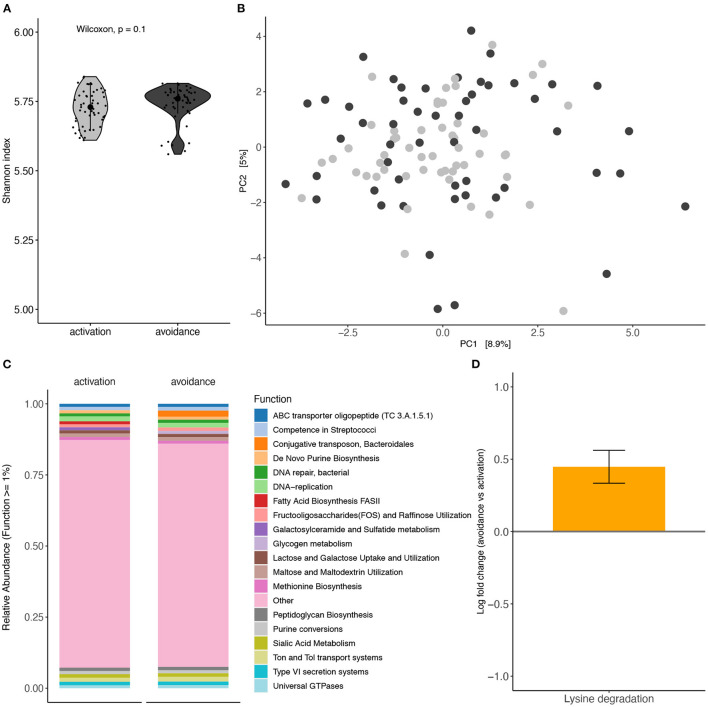
Functional profiling analysis of oral microbiota based on BADS. Functional profiling between BADS avoidance and activation groups **(A)** alpha diversity (Shannon index) and **(B)** beta diversity (Aitchison distance). **(C)** Average functional profiles with an abundance >1% are shown. **(D)** Lysine degradations different abundance between the two conditions (ANCOM-BC, q = 0.0692).

Functional profiling between both groups using shotgun metagenomics shows no significant differences with respect to alpha diversity (Shannon index; Mann-Whitney *U* test p = 0.1029) or beta diversity (Aitchison distance; PERMANOVA, *p* = 0.3862, *R*^2^ = 0.0076) (Figures 4A,B, respectively).

Average functional profile abundance for profiles with an abundance above 1% (19 profiles) is shown in Figure 4C. Lysine degradations, which are not among the top 19 profiles, showed a significantly different abundance between the two conditions (ANCOM-BC, q = 0.0692) (Figure 4D). Further, we did not detect a significant difference in GABA abundance between both groups (Supplementary Figure 3).

## Discussion

The oral microbiome represents an important gateway with unique composition and functional connections to the respiratory and gut microbiomes (6, 25). The gut microbiome supports a critical role in many health and disease conditions, including mood disorders (26). Therefore, we sought to explore the connections of the oral microbiome with depression (oral–brain axis).

In this study, we report for the first-time compositional and functional variations in the oral microbiome in healthy adults from local communities based on their BADS avoidance and activation score among cigarette smokers. Smoking is a major determinant of dysbiosis and inflammation that negatively impacts the overall oral health and microbial communities, evidenced by increased dental caries and periodontitis among cigarette smokers. We determined that the predominant phyla of *Firmicutes, Proteobacteria*, and *Actinobacteria*, likewise the most abundant genera, were *Streptococcus, Haemophilus D*, and *Veillonella*, and comparable to previous publications (27, 28). While there was a significant difference in alpha diversity between BADS avoidance and activation groups based on the Shannon index at the genus level, no significant differences in community composition using the Aitchison distance (beta diversity) were detected. *Bacteroidota, Campylobacterota, Firmicutes A, Firmicutes I*, and *Fusobacteriota* were significantly increased phyla in the avoidance group. However, *Verrucomicrobiota* were significantly diminished in the avoidance group. Remarkably, a study detected changes in *Verrucomicrobiota* relative abundance based on mice's adaptive immune system status, suggesting an intriguing link between immune status and the avoidance group (29, 30). Further differential abundance testing revealed 30 genera with significant enrichment differences between BADS avoidance and activation groups. Interestingly, *Fusobacterium* spp. were found to be associated with severe depression and anxiety among adolescents suggesting a possible targeted clinical intervention during this essential developmental period (3).

Next, network analysis of the top 50 genera displayed the highest variation between both groups. In contrast, network properties are not significantly different between groups in degree, betweenness centrality, and closeness centrality. However, we found that each network has specific hub nodes. *Campylobacter B, Centipeda*, and *Veillonella* were identified as hub nodes for the avoidance group, whereas *Haemophilus* and *Streptococcus* were identified as hub nodes in the activation group. Interestingly, *Veillonella* was found to be enriched in the oral microbiota of individuals with autism (31). However, another study reported that *Veillonella* was less abundant in the depressed cohort (27). Thus, despite the conflicting reports, *Veillonella* may produce metabolites that contribute to neurotransmitter dysfunction. For example, a study found that *Veillonella* spp. infections significantly increased soluble inflammatory mediators in the cervicovaginal microenvironment (32). Suggesting an intriguing link between *Veillonella*-induced pro-inflammatory mediators is consistent with the literature with regards to Autism.

Finally, we explored the metabolic potential of the shotgun metagenomic data. We identified 19 profiles with an average functional profile abundance above 1%, as shown in Figure 4C. Remarkably and consistent with previous literature, lysine degradations showed a significantly different abundance between BADS avoidance and activation groups. The oral microbiome of children with Autism reported taxa predominantly related to energy metabolism and lysine degradation pathways (33).

Mental illnesses such as anxiety and depression involve neurotransmitter dysfunction. In addition, the gut microbiome may produce neurotransmitters used in the human body, such as GABA, serotonin, and dopamine (34). Nevertheless, the amounts formed by bacteria are comparatively small and unlikely to impact human neurotransmission directly, but in combination with other factors, it might. Here, we did not detect a significant difference in GABA abundance between both groups, as shown in Supplementary Figure 3. Perhaps this is due to the confounding effect of cigarette smoking among the study population. Altogether, we acknowledge small sample limitation in this study, which needs further validation on a larger cohort and across populations with different environmental exposures and genetic backgrounds, gender and age variation and the use of other psychological assessment tools.

In conclusion, we performed a metagenomics analysis to explore the complex functional profiles of the oral microbiota in cigarette smokers based on the behavioral activation for depression scale from the Middle East. This data suggests that the oral microbiome may play an important role in mental health. The identified fluctuations within the oral microbiome and the BADS scores may suggest a biological pathway such as lysine degradations as an underlying linkage for the oral microbiome–brain axis, especially among cigarette smokers. Linking the dysbiotic *Veillonella* and *Fusobacterium spp* with lysine degradations among cigarette smokers provides new information that may eventually lead to novel and more effective clinical applications to better manage anxiety and depression. A more comprehensive study is warranted to explore the oral microbiome composition and functional interactions related to mental health and illnesses.

## Data Availability Statement

The raw sequencing data have been submitted to ENA and is publicly available under accession number PRJEB53577.

## Ethics Statement

The studies involving human participants were reviewed and approved by Research Ethics Committee at the University of Sharjah approved the study (REC-18-02-18-01). The patients/participants provided their written informed consent to participate in this study.

## Author Contributions

MAB, AK, ND, HA, and SI contributed to conception and design of the study. RA, RA-K, and MA collected data and performed initial analysis. AK and HB organized the database and performed the statistical analysis. MB wrote the first draft of the manuscript. HA, ND, AK, RA, RA-K, and MA wrote sections of the manuscript. All authors contributed to manuscript revision, read, and approved the submitted version.

## Funding

The University of Sharjah funded the project to Dr. MAB (Grant codes: 1901090253 and 1701090226).

## Conflict of Interest

The authors declare that the research was conducted in the absence of any commercial or financial relationships that could be construed as a potential conflict of interest.

## Publisher's Note

All claims expressed in this article are solely those of the authors and do not necessarily represent those of their affiliated organizations, or those of the publisher, the editors and the reviewers. Any product that may be evaluated in this article, or claim that may be made by its manufacturer, is not guaranteed or endorsed by the publisher.
