# Corrigendum: Altered composition of the oral microbiota in depression among cigarette smokers: A pilot study

**DOI:** 10.3389/fpsyt.2023.1175044

**Published:** 2023-03-16

**Authors:** Mohammad Tahseen Al Bataineh, Axel Künstner, Nihar Ranjan Dash, Rushud Mahmood Abdulsalam, Rafla Zaid Ali Al-Kayyali, M. Besher Adi, Habiba S. Alsafar, Hauke Busch, Saleh Mohamed Ibrahim

**Affiliations:** ^1^College of Medicine, University of Sharjah, Sharjah, United Arab Emirates; ^2^Department of Genetics and Molecular Biology, College of Medicine and Health Sciences, Khalifa University of Science and Technology, Abu Dhabi, United Arab Emirates; ^3^Center for Biotechnology, Khalifa University of Science and Technology, Abu Dhabi, United Arab Emirates; ^4^Lübeck Institute of Experimental Dermatology, University of Lübeck, Lübeck, Germany; ^5^Institute for Cardiogenetics, University of Lübeck, Lübeck, Germany; ^6^Department of Biomedical Engineering, Khalifa University of Science and Technology, Abu Dhabi, United Arab Emirates; ^7^Department of Mathematics, Khalifa University of Science and Technology, Abu Dhabi, United Arab Emirates

**Keywords:** avoidance, activation, BADS, metagenomics, oral microbiome

In the published article, there was an error in Figure 3 and Figure 4 as published. In the published article, the edges between nodes were not visible (Figure 3) and the figure/text was blurry (Figures 3, 4). The corrected Figure 3 and Figure 4 and their caption appear below.

**Figure 3:**
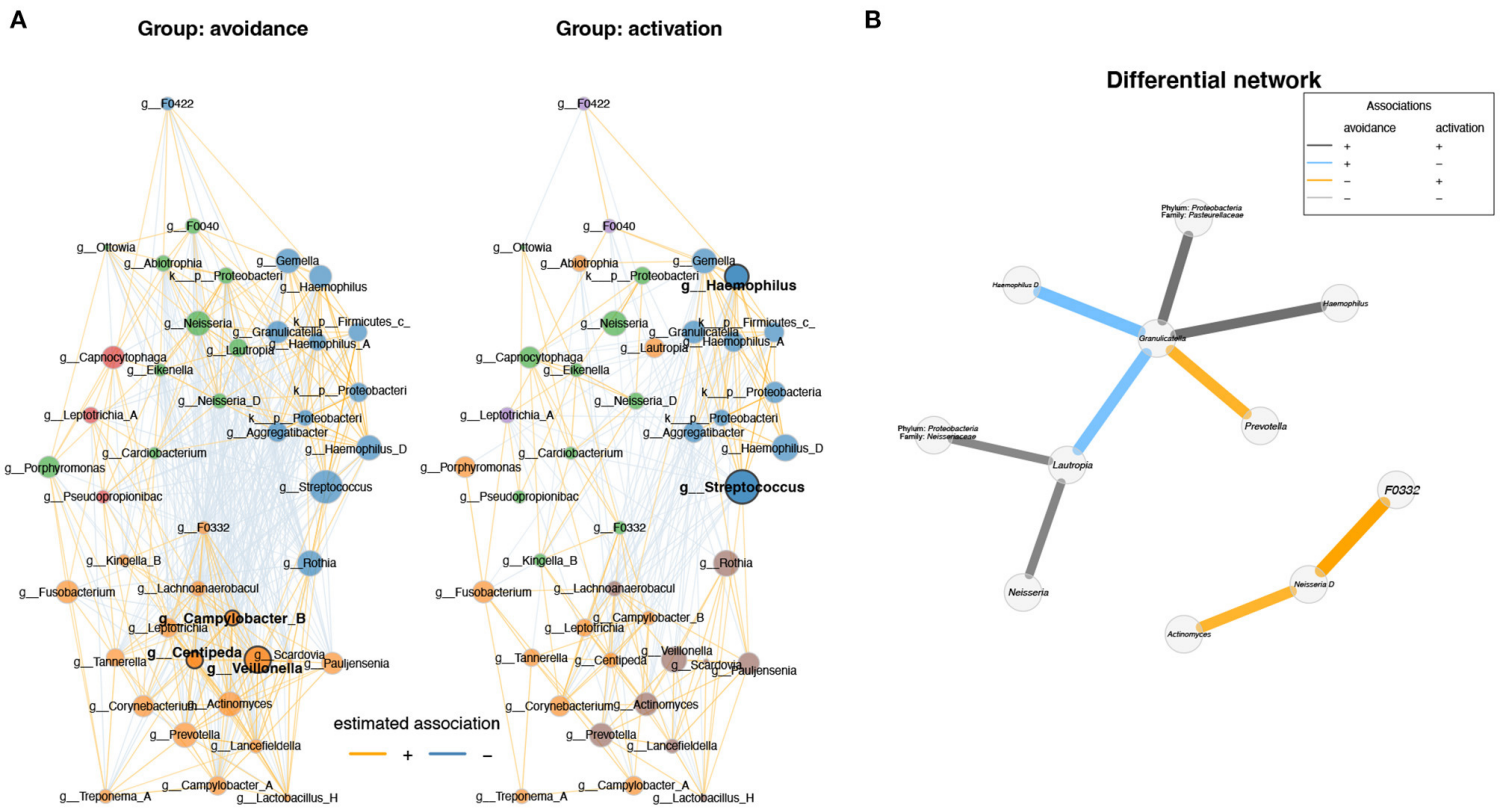
Differential network analysis between BADS avoidance and activation groups shows different correlations between genera. **(A)** Network analysis of the top 50 genera showing the highest variation between the two conditions and visualized the results (degree, betweenness centrality, closeness centrality; p > 0.05). Network specific hub nodes are shown in bold font. **(B)** The differential network shows the different correlations between genera (Fisher exact test, p-adj < 0.1 BH adjusted); if genus annotation was not available, phylum and family are shown.

**Figure 4:**
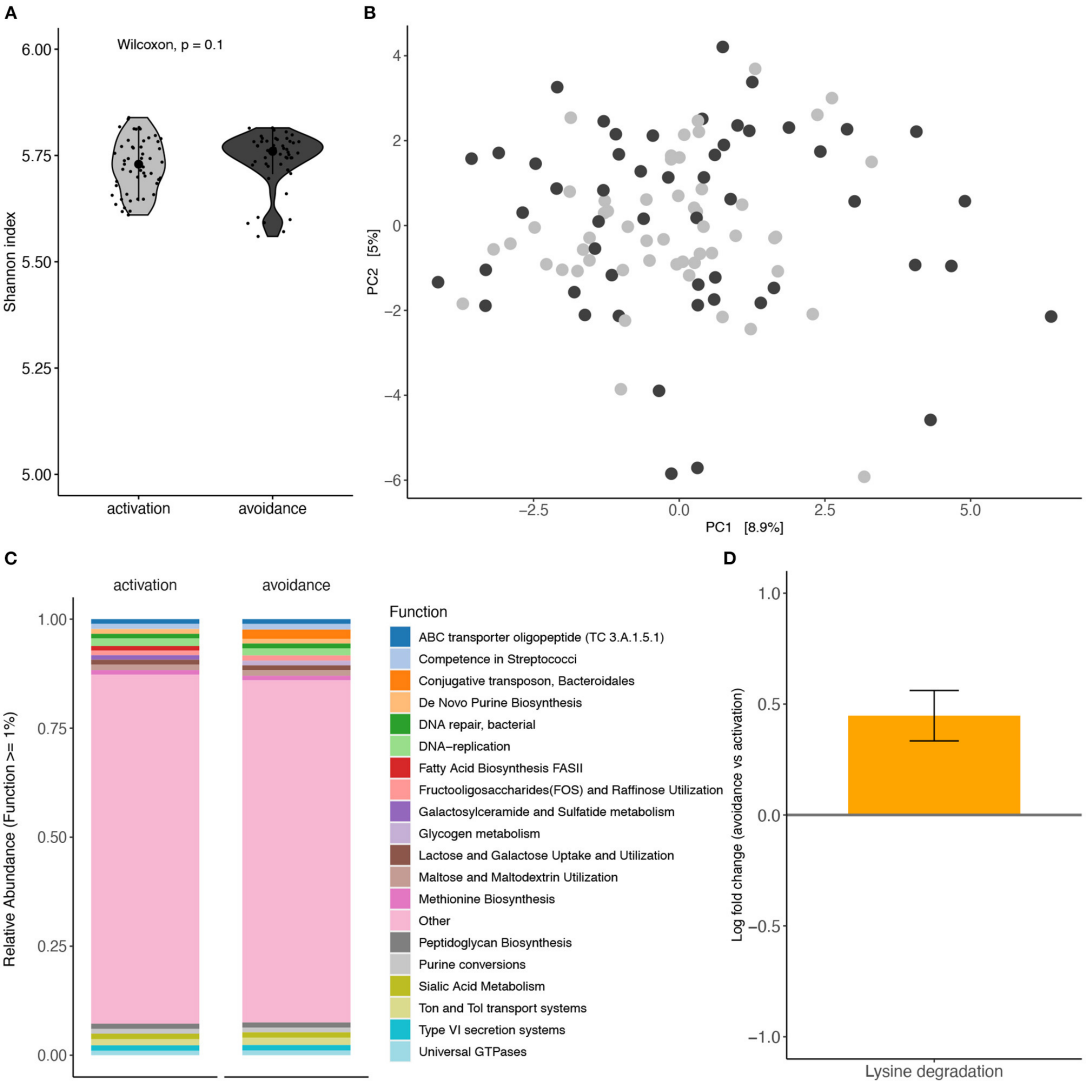
Functional profiling analysis of oral microbiota based on BADS. Functional profiling between BADS avoidance and activation groups **(A)** alpha diversity (Shannon index) and **(B)** beta diversity (Aitchison distance). **(C)** Average functional profiles with an abundance >1% are shown. **(D)** Lysine degradations different abundance between the two conditions (ANCOM-BC, q = 0.0692).

The authors apologize for this error and state that this does not change the scientific conclusions of the article in any way. The original article has been updated.

