# Supplementary figures and images for: Altered Composition of the Oral Microbiota in Depression Among Cigarette Smokers: A Pilot Study
Source: Front Psychiatry. 2022 Jul 19;13:902433. doi: 10.3389/fpsyt.2022.902433 (PMC9343996; doi:10.3389/fpsyt.2022.902433)

**FIG S1**

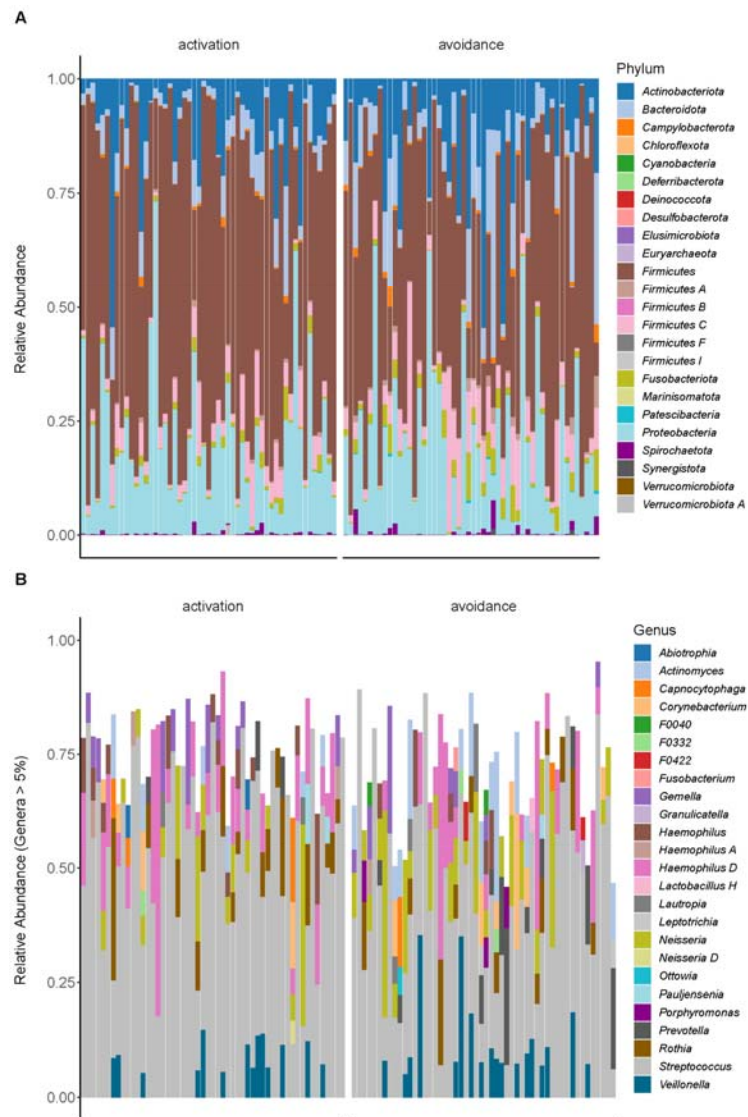

FIG S2

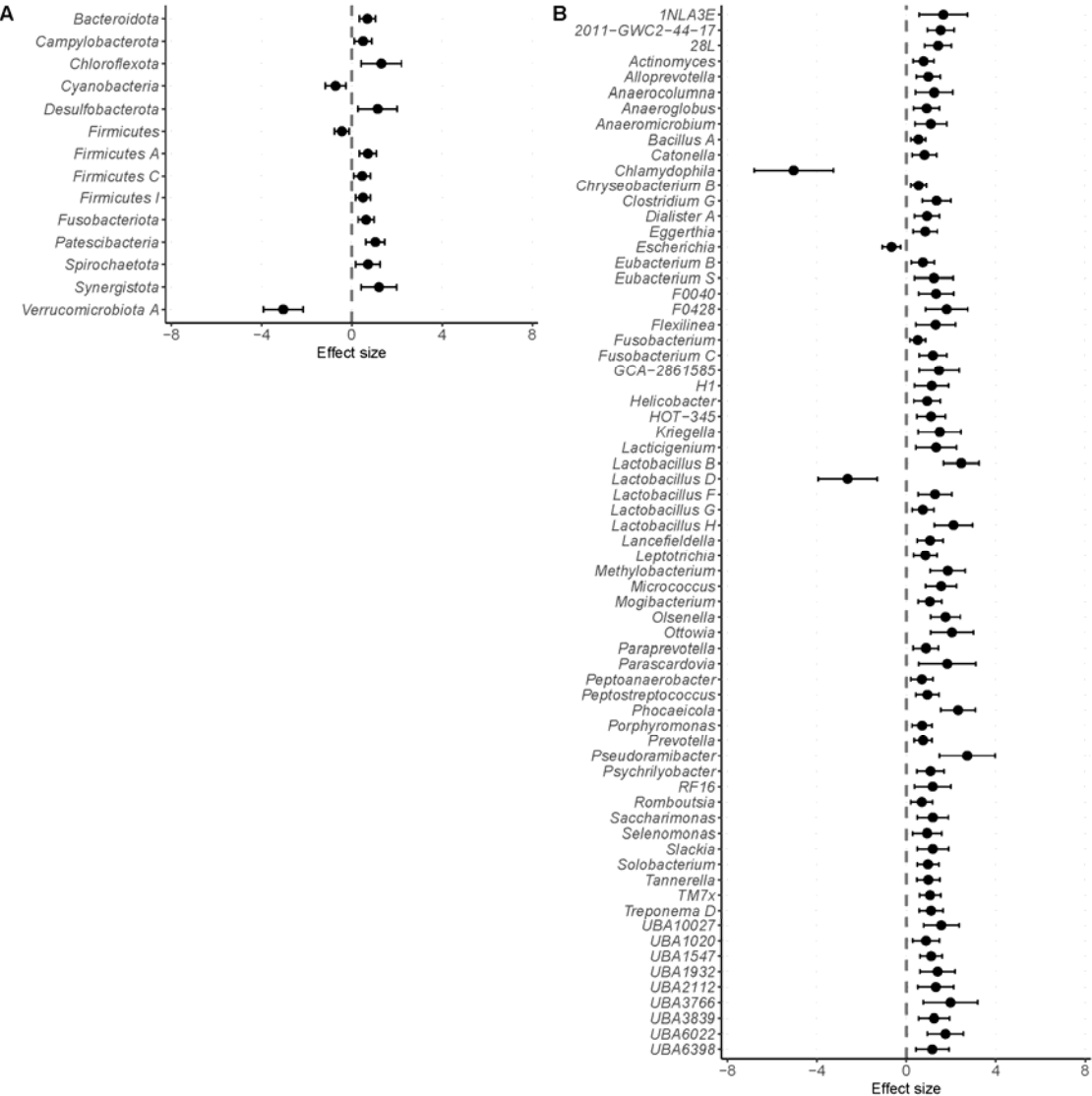

**FIG S3**

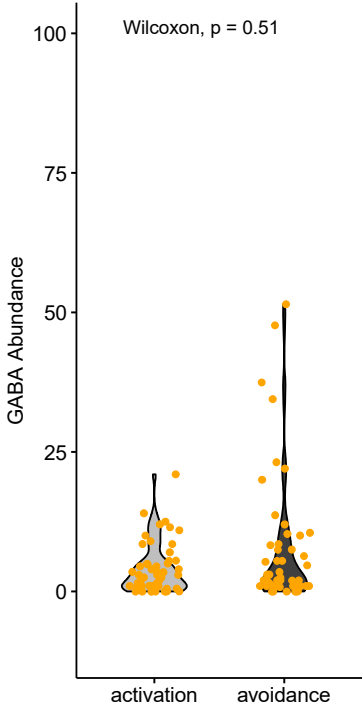

Supplement: Supplementary file 1 [file Data_Sheet_1.PDF]
